# Three-Dimensional Volumetric Assessment of Resected Gliomas Assisted by Horos Imaging Software: Video Case Series of Postoperative Tumor Analyses

**DOI:** 10.7759/cureus.13571

**Published:** 2021-02-26

**Authors:** Yasmeen Elsawaf, Stephanie Anetsberger, Sabino Luzzi, Samer K Elbabaa

**Affiliations:** 1 Medicine, University of Central Florida College of Medicine, Orlando, USA; 2 Pediatric Neurosurgery, Arnold Palmer Hospital for Children, Orlando, USA; 3 Department of Clinical-Surgical, Diagnostic and Pediatric Sciences, University of Pavia, Pavia, ITA

**Keywords:** glioma, volume analysis, horos software, osirix

## Abstract

Horos (LGPL 3.0; GNU Lesser General Public License, Version 3) is a free, open-source medical image viewer with a user-friendly interface and three-dimensional (3D) volumetric rendering capabilities. We present the use of Horos software as a postoperative tool for residual tumor volume analysis in children with high-grade gliomas (HGG). This is a case series of two pediatric patients with histologically confirmed high-grade gliomas who underwent tumor resection as definitive treatment from June 2011 to June 2019. Volumetric data and extent of resection were obtained via region of interest-based 3D analysis using Horos image-processing software. Horos software provides increased accuracy and confidence in determining the postoperative volume and is useful in assessing the impact of residual volume on outcomes in patients with high-grade gliomas. Horos software is a highly effective means of volumetric analysis for the postoperative analysis of residual volume after maximal safe resection of high-grade gliomas in pediatric patients.

## Introduction

Horos is a free, open-source medical image viewer and editing software [1]. Postoperative analysis of tumor resection has progressed from 2D MRI imaging to three-dimensional (3D) volumetric analysis with the use of neuronavigation technology. OsiriX is a well-known macOS-supported software that has been used for postoperative analysis since its development in 2003 [2]. A free, open-source medical imaging viewing alternative, Horos (Lesser General Public License (LGPL) 3.0) has gained recent notice in the neurosurgical community because of the familiar OsiriX-based interface and its useful 3D volumetric rendering capabilities [1]. There are currently no papers detailing the use of Horos technology for postoperative analysis. We provide a step-by-step video guide and case report of Horos software as a postoperative tool for residual tumor volume analysis in children with high-grade gliomas (HGG).

## Case presentation

Case #1

**Figure 1:**
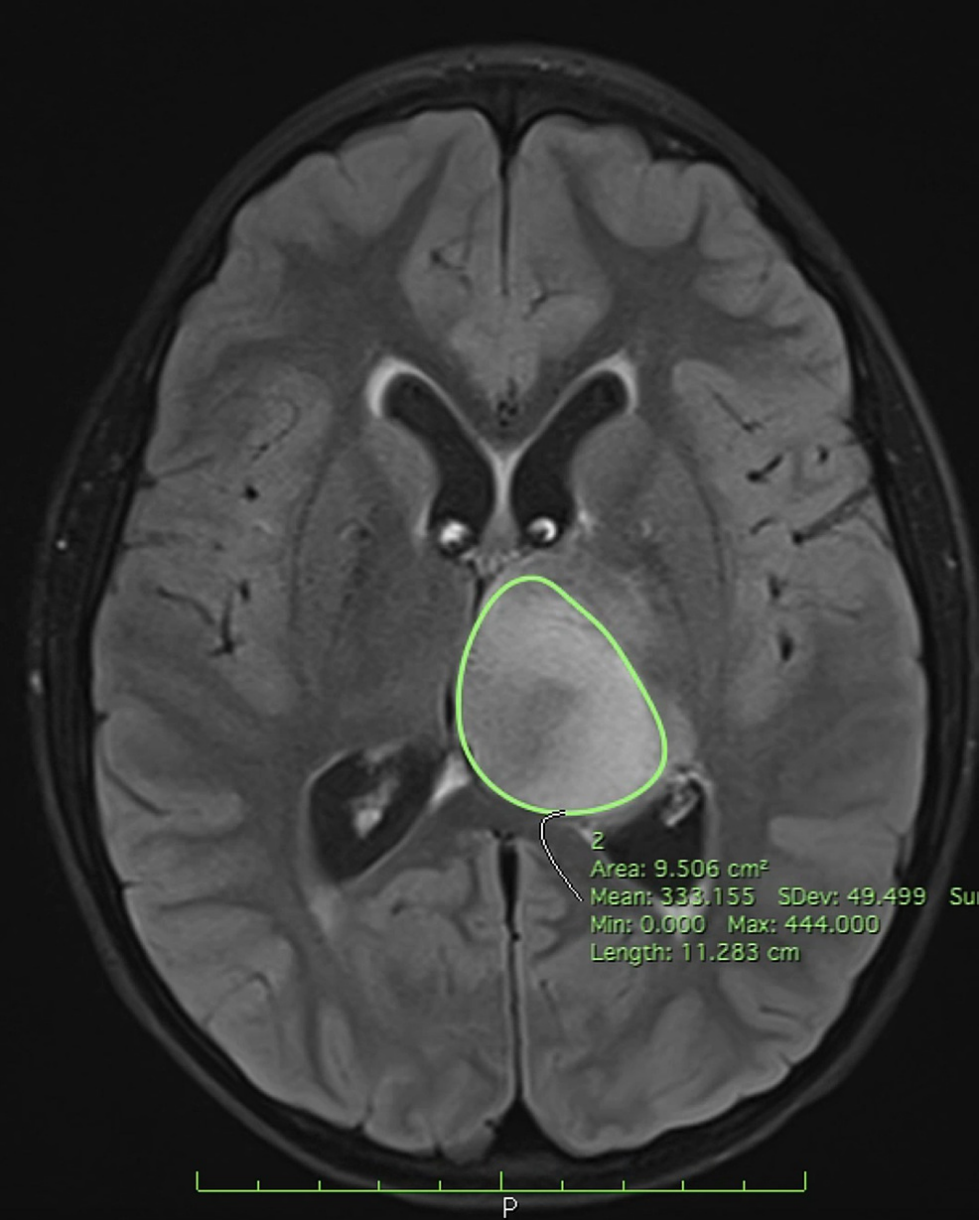
Preoperative T2 FLAIR MRI - Horos ROI segmentation Preoperative T2 FLAIR MRI demonstrating Horos region of interest (ROI) segmentation function with 3.6 cm x 3.4 cm x 4.8 cm mass of the left thalamus circumferentially outlined.

A nine-year-old male patient presented with a month of progressively worsening headaches and one week of emesis. The T2 FLAIR magnetic resonance imaging revealed a 3.6 cm x 3.4 cm x 4.8 cm mass of the left thalamus with an infiltrating nature and enhancement consistent with a glioblastoma multiforme (Figure 1). The patient underwent near-total resection and postoperative volume analysis was performed using Horos software. Preoperative tumor volume as measured by Horos software was 25.2048 cm^3^ and postoperative residual volume was 0.4805 cm^3^.

Case #2

**Figure 2:**
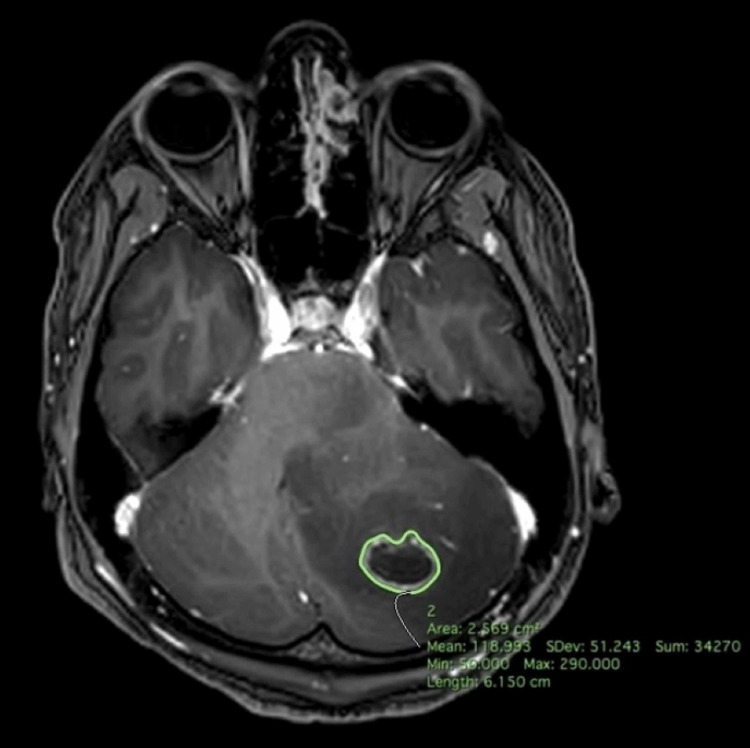
Preoperative T1-weighted MRI - Horos ROI segmentation Preoperative T1-weighted MRI demonstrating Horos region of interest (ROI) segmentation function with 4.2509 cm^3^ mass of the left cerebellum circumferentially outlined.

A 14-year-old male patient presented with one month of worsening medically irretractable headaches. The T1-weighted magnetic resonance imaging revealed a ring-enhancing mass of the left cerebellum consistent with a glioblastoma multiforme (Figure 2). The patient underwent near-total resection and postoperative volume analysis was performed using Horos software. Preoperative tumor volume as measured by Horos software was 4.2509 cm^3^ and postoperative residual volume was 0.1010 cm^3^.

Video 1 shows the Horos software guide.

**Video 1:** Tumor volumetric analysis guide: Horos software Video depicting volumetric analysis of high-grade glioma resection in the pediatric population using Horos software. Two case reports are presented with step-by-step instructions detailing the region of interest segmentation function, computing volume function, and 3D tumor volume rendering. Preoperative and postoperative tumor analysis are compared after maximal safe resection.

Preoperative and postoperative tumor volumetric analyses were performed per patient. Volumetric data and extent of resection were obtained via region of interest-based 3D analysis using Horos image-processing software. The image data utilized were digital imaging and communications in medicine (DICOM) files that were imported into the Horos program by CDs provided by the neuroradiology department. Horos version 3.3.6 (macOS 10.11+) was utilized (free download: horosproject.org/download-horos/) [1].

The interface of the program includes the database with the DICOM data in “Albums.” Once a sequence is selected, the 2D viewer allows viewing of the imaging series and editing functions with the dropdown tools. The region of interest (ROI) segmentation function can be selected as demonstrated in Video 1. In the ROI dropdown, “Closed Polygon Tool” should be selected, which will allow the user to outline the tumor on each image in the sequence. To outline the tumor using the “Closed Polygon Tool,” an ROI point can be placed by right-clicking the mouse at any point along the circumference of the tumor. The outline is completed by repeating this process and placing ROI points along the tumor in a clockwise or counterclockwise direction. Once outlined throughout most images in the series, the user can use the “Generate Missing ROIs” function to capture the entirety of the tumor within the MRI series and fill in any ROIs that were not manually placed. After this is complete, the user must rename all ROIs to the same name. This can be performed using the ROI dropdown menu and selecting “ROI Rename”. Once the rename box appears, the user can select the second button “All ROIs in this series” and rename appropriately. The “Compute Volume” function can be used to render a 3D image of the tumor with an accurate volume measurement. To visualize the tumor within the cranium in a 3D model, utilize the “3D window” dropdown menu and select "3D MIP." This will produce a 3D image of the patient’s cranium with the 3D tumor highlighted.

Preoperative and postoperative tumor volumes were analyzed to yield the percent extent of resection and residual volume.

## Discussion

Horos software is an easily accessible open-source software and effective tool for the postoperative analysis of residual volume after maximal safe resection of high-grade gliomas in pediatric patients. Horos provides increased accuracy and confidence in determining the postoperative volume and is useful in assessing the impact of residual volume on outcomes in patients with HGGs.

Similar software, such as OsiriX, has been utilized for imaging viewing and analysis of volumetric data; thus, OsiriX warrants comparison with the proposed Horos software (Table 1).

**Table 1:** Comparison: OsiriX versus Horos software FDA: Food and Drug Administration

Software	Source	32 vs 64-bit	Operating System	Customizable	FDA Approved
OsiriX	Closed-source (Lite with limited functionality)	32-bit Lite, 64-bit purchased	Mac	No	Yes - 64-bit purchased, No - 32-bit Lite
Horos	Open-source	64-bit	Both	Yes (custom licensed plugins)	No

OsiriX was initially marketed as an open-source project with conversion to a closed-source imaging viewer and analytic tool in 2015, which eventually prompted many users to switch to the easily attainable Horos project. OsiriX provides a 32-bit free trial “Lite” version, which runs up to 4.4 times slower than the 64-bit version. Horos offers a 64-bit processing speed to accommodate a larger number of image slices in an MRI series. Furthermore, Horos software is licensed under the GNU Lesser General Public License (GPL); this allows users to incorporate novel plugins into Horos software and establish legal protection for their original code. Horos also offers mobile compatibility with iOS-compatible devices and allows simultaneous connections to multiple medical imaging devices and processing systems. OsiriX allows for limited connections. Furthermore, OsiriX only runs on Mac (Apple Inc., Cupertino, CA) compatible operating systems and thus may pose limitations for customers running the software on with smaller hard drive storage capabilities such as the Macbook Air (Apple Inc., Cupertino, CA). Horos provides a convenient Horos Cloud account to allow for a hybrid environment that runs on Windows and Mac operating systems. Horos also provides an easily accessible web-based medical image viewer with limited analytic tools. Additionally, users of the OsiriX Lite software may encounter image overlays or watermarks that interfere with imaging utilization in publications or presentations, unless purchased. Due to the open-source model of Horos software, prohibitive image overlays are not engineered into the software [3-5].

Both Horos and OsiriX Lite (32-bit) are not FDA approved; however, the paid version of OsiriX is FDA-approved, which may be useful for patient cases in which FDA approval of analyzed data is required. In such a situation, a paid OsiriX subscription would be superior to the open-access Horos software [3].

Future applications of Horos software employing the described technique include the preoperative planning and postoperative analysis of non-glioma brain tumors as well as spinal tumors. Similar technologies, such as the aforementioned OsiriX software, have been utilized for these purposes [6-8].

## Conclusions

Horos is a free, open-source medical imaging viewer and analytic tool that offers ease of use and accuracy in the measurement of percent extent of resection and residual volume for pediatric high-grade gliomas. Compared to the OsiriX imaging viewing software, Horos provides easier access, compatibility with more operating systems, and a customizable interface to allow for enhanced speed and efficacy of analysis. The described volume rendering technique can be extended to a wide variety of intracranial and spinal masses.
